# Integrating implementation and user-centred design strategies to enhance the impact of health services: protocol from a concept mapping study

**DOI:** 10.1186/s12961-018-0403-0

**Published:** 2019-01-08

**Authors:** Alex R. Dopp, Kathryn E. Parisi, Sean A. Munson, Aaron R. Lyon

**Affiliations:** 1 Department of Psychological Science, University of Arkansas, 216 Memorial Hall, Fayetteville, AR 72701 USA; 2 Department of Human Centered Design and Engineering, University of Washington, 3960 Benton Lane NE, 428 Sieg Hall, Seattle, WA 98195 USA; 3 Department of Psychiatry and Behavioral Sciences, University of Washington School of Medicine, 1959 NE Pacific Street, Room BB1644, Box 356560, Seattle, WA 98195 USA

**Keywords:** Implementation strategies, User-centred design, Human-centred design, Concept mapping, Evidence-based practice

## Abstract

**Background:**

Innovative approaches are needed to maximise the uptake and sustainment of evidence-based practices in a variety of health service contexts. This protocol describes a study that will seek to characterise the potential of one such approach, user-centred design (UCD), which is an emerging field that seeks to ground the design of an innovation in information about the people who will ultimately use that innovation. The use of UCD to enhance strategies for implementation of health services, although promising, requires a multidisciplinary perspective based on a firm understanding of how experts from each discipline perceives the interrelatedness and suitability of these strategies.

**Method:**

This online study will use a combination of purposive and snowball sampling to recruit a sample of implementation experts (*n* = 30) and UCD experts (*n* = 30). These participants will each complete a concept mapping task, which is a mixed-method conceptualisation technique that will allow for identification of distinct clusters of implementation and/or UCD strategies. The research team has selected a set of implementation strategies and UCD strategies that each participant will sort and rate on dimensions of importance and feasibility. Data analyses will focus on describing the sample, identifying related clusters of strategies, and examining the convergences, divergences, and potential for collaboration between implementation science and UCD.

**Discussion:**

By leading to a better understanding of the overlap between implementation science and UCD, grounded within established theoretical frameworks, this study holds promise for improving the impact and sustainability of evidence-based health services in community settings.

## Background

Health services researchers have developed a number of evidence-based practices (EBPs) for the assessment of, intervention with, and management of medical and behavioural health conditions, yet these practices typically show reduced impacts in community settings and are rarely sustained for long periods following initial implementation [1, 2]. Numerous characteristics of EBPs can undermine their effectiveness in typical health service settings, including tensions between fidelity and flexibility during implementation, high complexity, and an assumption of unidirectional flow of expertise from researchers to practitioners (see [3] for a review). To maximise the public health benefits of EBPs, we need innovative approaches that can enhance their impact and sustainability through improved fit with a variety of health service contexts [4–6]. This protocol describes a study that will seek to characterise the potential that one such approach, user-centred design (UCD), has to provide a set of novel strategies that can enhance efforts to successfully implement EBPs in community settings.

Implementation science – an interdisciplinary field in the health sciences that is focused on improving the use of research evidence in everyday practice settings – has already identified a number of promising strategies for the implementation of EBPs over the past several decades. The most comprehensive review of these strategies is the Expert Recommendations for Implementing Change (ERIC) study, in which a panel of 35 implementation experts defined 73 discrete implementation strategies using the results of an earlier systematic review through a Delphi consensus-building process [7] and then sorted those strategies into nine conceptually distinct categories while also rating their importance and feasibility [8]. The ERIC study provided a much-needed common language and set of best-practice strategies used in implementation research and practice. However, close examination of the strategies reveals important gaps in the approach currently taken by the field. For example, when considering the multilevel domains specified in the Consolidated Framework for Implementation Research (CFIR) [9], most of the 73 strategies appear to focus on changes in the individuals and systems (inner/outer setting) that will adopt a health services innovation (e.g. ‘audit and provide feedback’, ‘conduct ongoing training’, ‘mandate change’) whereas only three seem to address the possibility of tailoring the innovation to local contexts (i.e. ‘develop and implement tools for quality monitoring’, ‘develop educational materials’, ‘promote adaptability’). Given the influence of EBP characteristics on key implementation outcomes such as acceptability, appropriateness and feasibility [10], as well as findings that modifications to EBPs are virtually guaranteed (and perhaps even desirable) in clinical practice [2, 11, 12], current approaches to the promotion of successful implementation are necessarily incomplete.

Recently, researchers have observed that fundamental EBP design problems – both in terms of practices themselves and the processes by which they are implemented – limit their adoption and sustained use in diverse health service settings [3]. Implementation of EBPs by health service providers and organisations often encounters significant challenges in terms of learnability and memorability (e.g. [13]), ease of use by intended users (e.g. [14]), and ability to address natural constraints of the destination context (e.g. [15]). Although some of these challenges could be addressed through improved attention to design during initial development of EBPs, some scholars have argued that EBPs are also frequently ‘over-designed’ in research settings – leading to inclusion of features that are not necessary or useful to end users – and instead recommended that healthcare practices be optimised within their ultimate implementation setting [16]. Recognising that the ERIC [7] compilation, while ground-breaking, speaks only sparingly to aspects of EBP design that may improve uptake, we suggest that there is a need for additional strategies that attend directly to those issues of design. To that end, it may be useful to seek innovative strategies from outside the health service fields and deepen our understanding of how multidisciplinary experts might collaborate to apply those strategies.

The field of UCD holds considerable potential for increasing the impact and sustainment of EBPs (see [3, 17, 18]). Drawing from research in human–computer interaction, industrial design, and cognitive psychology, UCD (and the closely related field of human-centred design) offers a set of strategies that seek to ground the design of an innovation in information about the people who will ultimately use that innovation [19–22]. Illustrative examples include identification of users and user needs, prototyping and rapid iteration, design simplification, and exploitation of natural constraints. The ultimate aim is to improve a product’s ‘usability in context’ by maximising effectiveness, efficiency, and satisfaction for specified users, goals, and activities [23]. The principles and strategies of UCD can be applied to the creation and improvement of software and physical products (e.g. [24]), service delivery (e.g. [25]), and training processes (e.g. [26]). UCD has most frequently been applied to the design of new health services and technologies (e.g. [18, 27, 28]), whereas applications to the delivery and sustainment of EBPs remain exceptional. Certain health service fields have yet to apply UCD extensively (e.g. behavioural health [3]), although there are a growing number of exceptions in the form of both intervention design studies (e.g. [29, 30]) and conceptual models (e.g. [16, 31]).

In sum, despite its potential, it remains unclear where UCD fits within the evolving landscape of implementation science and practice. Implementation frameworks have only recently begun to address the role of service design [16] and those efforts have been primarily conceptual rather than empirical. Thus, implementation experts have little guidance on how UCD fits within their existing perspectives and strategies regarding health services. As a first step in establishing such guidance, the Internet-based study described in this protocol will use concept mapping [32] to characterise the fit between implementation strategies (from ERIC [7]) and UCD strategies (from [19–22]) in the implementation of EBPs. We will identify and characterise clusters of implementation and UCD strategies – based on sorting and rating tasks completed by expert participants – in terms of their importance, feasibility and promise for interdisciplinary collaboration. Moreover, given that implementation is already a highly interdisciplinary and collaborative field, it seems reasonable to promote collaboration between implementation experts and UCD experts to capitalise on the promise of UCD for health services (rather than expecting implementation experts to develop expertise in UCD). Thus, as a secondary aim, we intend to describe the characteristics of professionals who are interested in EBP implementation and UCD as well as summarise recommendations by those experts regarding areas in which they require the most support for collaboration.

## Methods

### Participants

We will recruit implementation science experts (*n* = 30) and UCD experts (*n* = 30) to participate in this study. Expertise will be self-reported and can be based on experience in research, practice/industry, and/or education over the past 5 or more years. Our planned enrolment is twice the recommended sample size for concept mapping (*n* ≥ 15 per participant group; see [33]) to ensure that we can make meaningful comparisons between results from implementation versus UCD experts.

### Concept mapping

It is challenging to systematically represent the relationships that individuals perceive between various concepts or ideas (such as implementation strategies and UCD strategies). We will address that challenge through the use of concept mapping [32], a methodological approach that guides participants through a structured conceptualisation process where each participant sorts ideas into groups that represent their interrelationships and then rates the ideas on key dimensions. Researchers in health services (e.g. [34, 35]) and implementation science (e.g. [8, 36]) regularly use concept mapping because it is a self-contained mixed-method approach that allows for sequential data collection, explanation/elaboration, and revision to questions and concepts. Specifically, concept mapping involves collection of qualitative (sorting) and quantitative (rating) data that are subsequently analysed to produce concept clusters. Determination of the final set of concept clusters again involves a combination of quantitative (generation of empirically derived sets of clusters) and qualitative (selection of the appropriate set of clusters based on conceptual clarity and credibility) approaches. Moreover, concept mapping can produce reliable and valid results from small samples [33], making it especially feasible as a mixed-method approach.

The concept mapping process consists of four phases, namely (1) idea generation, (2) sorting, (3) rating, and (4) analysis (see [32]). We will use Concept Systems Global MAX (CSGM) [37], a secure, confidential web-based software platform, to conduct each of those steps in this study.

### Idea generation

The first step of concept mapping involves the generation of the ideas or concepts that participants will sort and rate [32]. We completed idea generation as a research team by using existing resources that documented implementation and UCD strategies to generate a comprehensive list of 66 strategies (36 implementation, 30 UCD). The third and fourth authors served as expert consultants regarding UCD and implementation strategies, respectively, but all team members reviewed the full list of strategies. Each strategy has a short name as well as a more comprehensive, yet still brief (< 50 words), definition. Tables 1 and 2 present the full list of strategies; a list of the definitions is available from the first author upon request. After finalisation of the lists of strategies, we uploaded each strategy into CSGM as a separate ‘statement’ for subsequent sorting and rating by participants.

**Table 1:** List and characteristics of implementation strategies generated for sorting and rating

Implementation strategy	ERIC results^a^		Primary CFIR domain(s)^b^			
	Importance	Cluster	OS	IS	ID	IN
Access new funding	3.57	F	X			
Alter incentive/allowance structures	3.17	F	X	X		
Alter patient/consumer fees	2.60	F	X	X		
Assess for readiness and identify barriers and facilitators	4.60	E	X	X	X	
Audit and provide feedback	4.40	E			X	
Build a coalition	3.77	S	X	X		
Centralise technical assistance	2.73	I	X			
Change accreditation or membership requirements	2.17	CI	X			
Change record systems	2.83	CI		X		
Conduct local consensus discussions	3.63	S		X		
Conduct ongoing training	4.17	T			X	
Create or change credentialing and/or licensure standards	2.23	CI	X			
Develop and implement tools for quality monitoring	4.37	E				X
Develop and organise quality monitoring systems	4.33	E		X		
Develop educational materials	3.80	T				X
Develop resource sharing agreements	3.07	CL	X			
Facilitate relay of clinical data to providers	4.17	CL		X		
Facilitation	4.13	I		X	X	
Fund and contract for the clinical innovation	3.67	F	X			
Identify and prepare champions	4.20	S			X	
Increase demand	3.30	CO	X			
Involve patients/consumers and family members	3.87	CO			X	
Mandate change	3.23	CI	X	X		
Obtain formal commitments	3.77	S	X			
Organise clinician implementation team meetings	3.97	S		X		
Place innovation on fee for service lists/formularies	3.40	F	X			
Promote adaptability	3.90	A				X
Provide local technical assistance	3.97	I		X		
Provide ongoing consultation	4.17	T			X	
Purposefully re-examine the implementation	4.40	E		X		
Recruit, designate, and train for leadership	3.93	S		X		
Remind clinicians	3.23	CL		X		
Tailor strategies	4.37	A		X		
Use data experts	3.23	A	X	X		
Use train-the-trainer strategies	3.33	T	X	X		
Work with educational institutions	2.73	T	X			

*ERIC* Expert Recommendations for Implementing Change, *CFIR* Consolidated Framework for Implementation Research, *OS* outer setting, *IS* inner setting, *ID* individual, *IN* intervention, *X* strategy primarily targets that domain.

^a^Data taken from the ERIC study concept mapping results [8]; importance and feasibility ratings range from 1.00 (lowest) to 5.00 (highest); abbreviations for clusters are as follows: *E* use evaluative and iterative strategies, *I* provide interactive assistance, *A* adapt and tailor to context, *S* develop stakeholder interrelationships, *T* train and educate stakeholders, *CL* support clinicians, *CO* engage consumers, *F* utilise financial strategies, *CI* change infrastructure.

^b^As assigned by the first and third authors through a consensus-building discussion

**Table 2:** List and primary sources of user-centred design strategies generated for sorting and rating

User-Centred Design Strategy	Primary source(s)^a^			
	OUE [19]	UMD [20]	CD [21]	FG [22]
Apply process maps to system-level behaviour	X			
Apply task analysis to user behaviour	X			
Build a user-centred organisational culture	X			
Collect quantitative survey data on potential users	X			
Conduct artifact analysis		X		
Conduct co-creation sessions				X
Conduct competitive user experience research	X			
Conduct design charrette sessions with stakeholders		X		
Conduct experience sampling	X	X		
Conduct focus groups about user perspectives	X			
Conduct heuristic evaluation		X		
Conduct interpretation sessions with stakeholders	X			
Conduct interviews about user perspectives	X			
Conduct observational field visits	X			
Conduct usability tests	X	X		
Define target users and their needs	X			
Define work flows		X		
Design in teams			X	
Develop a user research plan	X			
Develop experience models	X			
Develop personas and scenarios	X	X		
Engage in cycles of rapid prototyping		X		X
Engage in iterative development	X	X		
Engage in live prototyping				X
Examine automatically generated data	X	X		
Prepare and present user research reports	X			
Recruit potential users	X			
Use associative object-based techniques	X	X		
Use dialogic object-based techniques	X			
Use generative object-based techniques	X			

^a^The numbers in brackets refer to the citation for each source in the reference list.

*OUE* Observing the User Experience, *UMD* Universal Methods of Design, *CD* Contextual Design, *FG* Field Guide for Human-Centred Design, *X* strategy is described in that source

### Selection of implementation strategies

We selected a representative subset of implementation strategies from the full list of ERIC strategies [7] for inclusion in the present study using a three-step process. First, we gathered data to help us determine representativeness of our selections in terms of the implementation process level(s) involved. To that end, the first and fourth authors reviewed all 73 strategies and determined, through a process of discussion and consensus, which CFIR domains [9] each strategy targeted. Although it is possible to target any CFIR domain with any ERIC strategy (see [38]), we focused on the domains that were most likely to be targeted or represented the best fit to cut down the 2847 possible combinations. We determined that 32 strategies primarily targeted the outer setting (i.e. extra-organisational factors), 34 primarily targeted the inner setting (i.e. intra-organisational factors), 18 primarily targeted individuals involved in implementation, and three primarily targeted the intervention. The full list of CFIR domain assignments is available from the first author upon request.

Second, using the above information, we selected strategies from each of the nine clusters of implementation strategies (e.g. use evaluative and iterative strategies, provide interactive assistance) developed from a concept mapping exercise as part of the ERIC study [8]. We selected strategies within each cluster according to the following decision rules: (1) select the strategies with the highest importance ratings (i.e. to focus on the most useful strategies, regardless of their feasibility); (2) to roughly reflect the proportion of strategies across CFIR domains (see previous paragraph), include two strategies that target the outer setting, two that target the inner setting, one that targets individuals, and one that targets the intervention whenever possible; and (3) if a strategy is categorised under multiple CFIR domains, count it under the domain that occurs least frequently in that cluster (or across all clusters in the event that multiple domains are tied for lowest frequency in a cluster). This initial selection approach resulted in a set of 36 implementation strategies, but after reviewing those strategies, we noticed that our approach tended to exclude relatively high-importance strategies from larger clusters in favour of low-importance strategies from smaller clusters. Thus, finally, to correct for that tendency, we modified the decision rules such that, within each cluster: (4) if more than 75% of strategies from a given CFIR domain are selected, remove strategies (starting with the lowest importance) until coverage of that domain is ≤ 75% but ≥ 25%; and (5) if less than 25% of strategies from a given CFIR domain are selected, select additional strategies (starting with the highest importance) until coverage of that domain is ≥ 25% but ≤ 75%. These modified decision rules removed three strategies and subsequently added three strategies, respectively, resulting in a final set of 36 implementation strategies that were representative across clusters (i.e. 35–75% of strategies from each cluster) and CFIR domain (i.e. 33–100% of strategies from each domain) while focusing on strategies with the highest importance ratings. Table 1 lists the importance rating, cluster, and CFIR domain(s) for each selected strategy.

### Selection of UCD strategies

UCD is a diverse, innovative field that remains highly variable in terms of language and approaches. As such, no comprehensive list of UCD strategies (i.e. comparable to the ERIC study) currently exists. Instead, we selected strategies for inclusion in the present study from a variety of commonly used UCD resources that were recommended by the third author (i.e. [19–22]). After an initial review of each resource, we determined that Observing the User Experience (Goodman et al. [19]) most closely matched the ERIC project in terms of the conceptual level at which it described strategies (i.e. the other resources were either too broad or too detailed in the way that they described UCD strategies). Therefore, we primarily selected UCD strategies from Goodman et al. [19], but also supplemented those selections with unique, complementary strategies from the other resources. The first and second authors initially reviewed each resource and extracted strategies, including drafting of names and definitions, after which the third author reviewed the strategies. All three of those authors then engaged in a process of discussion and consensus until they agreed on a final list of 30 strategies that provided a comprehensive overview of the current major approaches to UCD. Table 2 lists those strategies as well as the primary source(s) for each strategy; more details are available in a separately published glossary of UCD strategies [39]. Two-thirds of the strategies were from Goodman et al. [19], whereas the other resources contributed the remaining strategies and also described 27% (6 out of 22) of those from Goodman et al. [19].

### Procedures involving participants

**Fig. 1:**
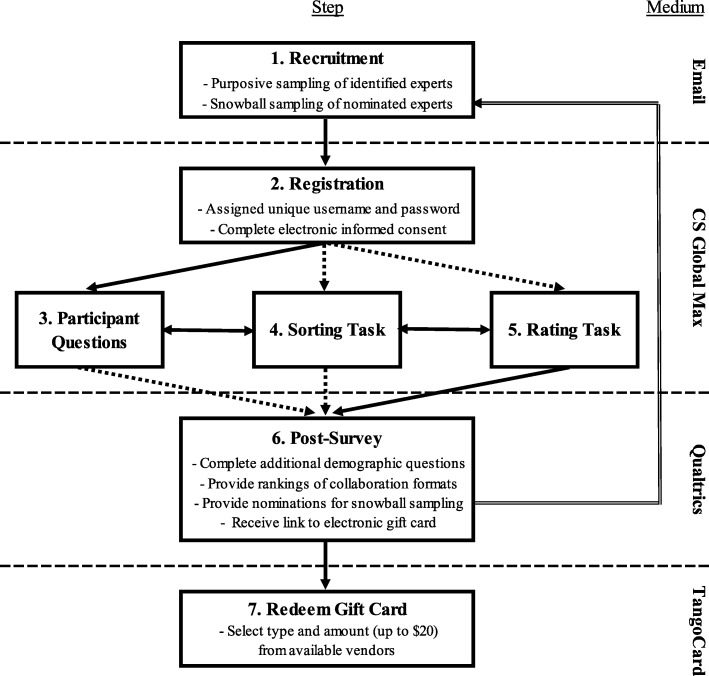
Flow chart of participant activities by step (1–7). Solid arrows represent the typical sequence of step completion in the study. Dashed arrows represent the fact that participants can initiate and complete steps (3) through (5) in any order. Double-sided arrows represent the fact that participants can stop and start steps (3) through (5), and may switch between those tasks, as often as they choose. The curved arrow represents feedback of the nominations in step (6) back to recruitment in step (1). Dashed lines (no arrowheads) separate activities that are completed via different mediums, with the medium for each activity listed on the right side of the figure

Recruitment and data collection for this study began in February 2018. Figure 1 provides a flow chart of the activities involving participants in the study. Over the course of the study, participants will move through seven steps, as follows: (1) recruitment, (2) registration, (3) answering participant questions, (4) completion of sorting task, (5) completion of rating task, (6) filling out an anonymous post-survey, and (7) redeeming a $20 electronic gift card as compensation. All procedures will be completed via the Internet using four different services or websites. Step (1) will be completed via email. Steps (2) through (5) will be completed in CSGM. The CSGM system allows participants to complete steps following registration (i.e. 3–5) in any order, although we designed the flow of steps within our study to encourage participants to complete the steps in the order listed above. Participants can also stop and start the activities as often as they wish. Step (6) will be completed in Qualtrics, a secure, confidential web-based survey collection platform. Step (7) will be completed via TangoCard, a website that provides participant incentives in the form of electronic gift cards. We expect that completion of all steps will take approximately 60 min. Participants will be free to withdraw from the study at any time, or skip any questions that they do not wish to answer, without penalty to them.

### Recruitment

To ensure our participants have appropriate expertise and constitute a nationally representative sample, recruitment will proceed through a combination of purposive and snowball sampling [40]. We will first employ purposive sampling by sending invitation emails to experts in implementation science and/or UCD from research centres and professional organisations that are centres of excellence for research in both implementation science and UCD; these centres will be identified with the assistance of the expert consultants (third and fourth authors). Then, we will engage in snowball sampling by asking each participant that completes the study to nominate up to five potential participants for invitation via email.

### Registration

Interested participants will contact the study coordinator (second author) and will receive a follow-up response email that contains login information for the CSGM web-based platform. Each participant will be assigned a unique, randomly generated username and password for CSGM; that username will also serve as their participant ID for the study. Once they log into CSGM, the participant will need to read and electronically sign the informed consent form for the study before they can begin participation. As in the ERIC study, we will retain and analyse data for all participants, including those who do not complete every study step.

### Participant questions

Participants will report on key demographic variables in CSGM. Importantly, each participant will self-identify their primary professional expertise as ‘implementation expert’ or ‘UCD expert’; we expect that some participants will have expertise in both areas but we will ask each to choose a primary area. Responses to this question will subsequently be used to compare the responses of implementation versus UCD experts. Four other questions will collect descriptive information and also serve as prompts to help participants reflect on professional experiences that are relevant to the sorting and rating tasks. Specifically, participants will report how many years of experience they have in their area of expertise; the percentage of their time spent on implementation and/or UCD activities that involved interdisciplinary collaboration; which primary service sectors (e.g. healthcare, education, human services, prevention and health promotion) are the focus of their work; and which system level they primarily seek to influence through their work (i.e. the CFIR [9] intervention, individual, inner setting, and outer setting domains). CSGM only allows for five participant questions, so additional demographic data will be collected in the post-survey instead.

### Sorting and rating

The middle two phases of concept mapping, namely sorting and rating [32], occur in tandem and will involve collecting input from a sample of individuals with content expertise through the CSGM platform. For the sorting step, participants will sort virtual cards that represent the 66 implementation and UCD strategies into groups. Each card will present the name of a strategy, followed by its definition; longer definitions will not be fully displayed in standard view, but the participant can click a button on the card that will expand it and display the full definition. The order of card presentation will be randomised, with no distinction made between implementation versus UCD strategies. Participants will be instructed to group the strategies according to their view of their meaning or theme and to give each group a name that describes its theme or contents; approximately 5 to 20 groups are expected given the number of strategies to be sorted. Participants will be instructed to not create groups according to priority (e.g. ‘First Steps’) or value (e.g. ‘Important’), to avoid catch-all groups (e.g. ‘Miscellaneous’), and to sort all of the cards even if that means creating groups that only contain one card.

For the rating step, participants will rate each strategy on its importance and feasibility on a scale ranging from 1 (least important/feasible) to 5 (most important/feasible). The strategies and their definitions will be presented in list format during this step, with the rating scale next to each strategy, and the list will be prefaced by the following instructions: “Please select a number from 1 to 5 for each discrete strategy to provide a rating in terms of how important (feasible) you think it is for the improvement of health and social services. Keep in mind that we are looking for relative importance (feasibility), and use all the values in the rating scale to make distinctions.” Our rating dimensions and instructions were adapted from the ERIC study [8].

### Post-survey

After completing all steps in CSGM, the system will display a link to a post-survey in Qualtrics. The participant will use that survey to provide anonymous information that is not linked to their CSGM account. Specifically, they will be asked to provide additional demographic information (i.e. gender, race/ethnicity); rate the top three areas in which support is needed to increase collaboration between implementation experts and UCD experts, by selecting from a list of seven options generated by the study authors (e.g. meeting colleagues from the other disciplines, securing funding for collaborative projects, developing a collaborative project idea or blueprint) and/or suggesting other areas where support is needed (i.e. up to three free-response options); and nominate up to five colleagues to participate in the study (i.e. by providing name, email address, and area of expertise).

### Gift card redemption

On the last page of the post-survey, participants will receive a unique link from TangoCard that allows them to select a $20 gift card from a variety of available vendors (e.g. Amazon, iTunes, Starbucks). For Qualtrics to display the TangoCard link, participants must enter their CSGM username on an authentication screen; the information input into the authentication screen is not stored by the Qualtrics system and is not visible to the research team, but it is simply used to link the participant to their designated gift card link.

### Analytic strategy

After sorting and rating are complete, the final step of concept mapping is data analysis [32]. In addition to analyses of concept mapping data, we will use software programmes such as Excel and SPSS to examine descriptive statistics (e.g. means, standard deviations, frequency counts) for demographic variables and survey responses (e.g. ratings of collaboration options). As noted previously, we will analyse data provided by all participants (including those who do not complete all study steps), which could result in uneven numbers of implementation experts versus UCD experts for certain analyses. This is not a problem in concept mapping because the technique does not rely on traditional assumptions for statistical significance and power [33].

The analytic strategy for concept mapping data requires further explanation. To start that process, we will use multidimensional scaling techniques (embedded in CSGM [37]) to identify clusters of implementation and UCD strategies that were generated most consistently across participants. CSGM will empirically generate any number of clusters that the researcher specifies, but it is up to the researcher to select the appropriate set of clusters (e.g. there is no established cut-off for minimum bridge values, which represent the closeness of relationships between concepts in a cluster, that indicate ‘good’ clusters). Consistent with the ERIC study [8], the research team will review the results for conceptual clarity and credibility before selecting which set of clusters to report in our findings. To guide that process, we will examine visual summaries of the data that can be produced in CGSM, such as cluster maps, which represent the relatedness of concepts within and between clusters in terms of visual distance and can be weighted by key dimensions (e.g. importance, feasibility), and ladder graphs, which provide a visual representation of the relationship between dimensions (e.g. importance and feasibility) within and across clusters. We will also consider the extent to which clusters are consistent with or expand upon the (1) clusters of implementation strategies identified in the ERIC study [8], (2) CFIR domains [9], and (3) the Promoting Action on Research Implementation in Health Services framework [41], which describes implementation of research evidence in practice contexts.

After we have selected the final set of clusters, we will characterise the importance and feasibility ratings for each cluster and strategy. We will use CGSM to visually represent the relationships among concepts using go-zone graphs that plot clusters and strategies along key dimensions (e.g. plots of importance vs. feasibility). Go-zone graphs using construct means as graph axes can be used to characterise ‘Go/No-Go/Proceed-With-Caution’ zones among the four quadrants of a scatterplot; these zones will illustrate which clusters are particularly promising for future actions such as collaboration among implementation experts and UCD experts. Moreover, to promote deeper understanding of the connections between implementation and UCD, we will also explore the level of convergence (i.e. extent to which concepts represent overlap between fields) and divergence (i.e. extent to which concepts represent unique contributions from a single field) in our findings by inspecting the number and types of strategies in each cluster.

Following the aforementioned initial analyses of concept mapping data, which will examine results across rating and sorting data from all participants, we will also examine results separately for each subgroup of experts (i.e. implementation vs. UCD). Specifically, we will apply the same analytic approach described previously with data separated by professional expertise, and we will examine differences in (1) the number and contents of clusters and (2) the ratings of each cluster. Within CSGM, we will generate the same data visualisations (e.g. concept maps, ladder graphs, go-zone graphs) separately for each group. Outside of CGSM, we will use multivariate general linear models to examine between-group differences in average ratings of importance and feasibility across professional expertise and clusters, and we will also use χ^2^ analyses to examine differences between professions in the amount of convergence and divergence in clusters.

## Discussion

This mixed-methods online study will explore the convergence, divergence and potential for interdisciplinary collaboration among implementation strategies and UCD strategies. This study will use innovative technology to engage experts from multiple professional backgrounds (i.e. implementation experts and UCD experts) in a structured conceptualisation process. Specifically, a web-based concept mapping platform will be used to capture how experts from these two disciplines conceptualise the relationships between implementation and UCD strategies, view their importance and feasibility, and converge or diverge in their perspectives. The virtual nature of the study and its low time commitment (i.e. approximately an hour) decreases the logistical barriers to obtaining involvement from expert stakeholders. Furthermore, the ability to organise these strategies into meaningful groups is essential because inclusion of UCD strategies otherwise has the potential to greatly increase the already high number of available implementation strategies (i.e. 73 in the ERIC project). Uptake of UCD strategies in health services is unlikely, even if they are viewed as important and feasible by experts, unless those strategies are integrated into existing frameworks for health services research and practice.

More specifically, the results of the present study will inform our perspectives on the potential for integrating UCD into implementation science in several ways. First, the clusters of strategies generated will deepen our understanding of how UCD strategies relate to traditional implementation strategies. For example, it may be ideal for implementation and UCD experts to work side-by-side to execute strategies from more convergent strategy clusters (i.e. several implementation and UCD strategies that focus on the same or related goals), whereas experts might better work sequentially or in parallel for more divergent clusters (i.e. groups of implementation or UCD strategies that have little direct overlap with strategies from the other discipline). Second, data on the importance and feasibility of clusters and strategies will be used to characterise their promise for interdisciplinary integration. For instance, we will prioritise clusters and strategies in the ‘Go’ zone (high importance and feasibility) of the Go-zone graph in future work seeking to promote collaboration, whereas strategies and clusters in the ‘No-Go’ zone (low importance and feasibility) will be de-emphasised. Third, to the extent that perspectives differ between implementation experts and UCD experts, we will use that information to identify areas of particular challenge in bridging perspectives across these two disciplines. Additional research may be necessary to resolve discrepancies; for example, if experts in one discipline view the strategies as more divergent or less important than do experts in the other, it will be important to understand the reasons for those discrepancies and refine our conceptualisation of the strategies accordingly. Finally, the post-survey questions about supporting collaboration will help identify potentially useful approaches for joining implementation experts and UCD experts together so that they can put into practice the interdisciplinary perspectives identified in this study. Specific approaches to collaboration will also likely merit additional investigation as we move this line of research forward.

Assuming that the results of the concept mapping process support the potential benefits of UCD in the implementation process, as we anticipate, this study will help expand the scope of interdisciplinary collaboration within implementation science and practice. In the future, our research team plans to explore the potential of resources (e.g. mentored development programmes) and tools (e.g. shared online workspaces) – informed by the results of this study – to support collaboration around the design of EBPs and related implementation supports. Such resources and tools would complement the limited, but growing, set of implementation training initiatives that are currently available to the field [42].

## Abbreviations

CFIR: Consolidated Framework for Implementation Research
CSGM: Concept Systems Global MAX
EBP: evidence-based practice
ERIC: Expert Recommendations for Implementing Change
UCD: user-centred design
